# Size-resolved Pb distribution in the Athabasca River shows snowmelt in the bituminous sands region an insignificant source of dissolved Pb

**DOI:** 10.1038/srep43622

**Published:** 2017-03-06

**Authors:** Muhammad Babar Javed, Chad W. Cuss, Iain Grant-Weaver, William Shotyk

**Affiliations:** 1 Department of Renewable Resources, University of Alberta, AB Canada T6G 2H1, Canada

## Abstract

Lead (Pb) is a metal of special importance because of its long history of commercial and industrial use, global atmospheric contamination accelerated by the use of gasoline additives, and health effects, with children being especially vulnerable. Global atmospheric Pb pollution reached its zenith in the 1970’s, but subsequent impacts on freshwater aquatic systems are poorly understood. Employing metal-free sampling and handling protocols, we show that snowmelt from the Athabasca bituminous sands region is an insignificant source of dissolved Pb to the Athabasca River (AR). Total Pb in the AR is low, and almost entirely in particulate form. Lead in the suspended solids in the AR exactly follows thorium (Th), a conservative lithophile element, and a linear regression of Pb against Th (Pb = 1.6 × Th + 0.0; R^2^ = 0.99) yields a slope identical to the Pb/Th ratio in the Upper Continental Crust. In the “dissolved” fraction, the Pb/Th ratio is equivalent to that of deep, open ocean seawater; and dominated by colloidal forms. Taken together, these results show that the efforts of recent decades to reduce anthropogenic Pb to the environment have been successful: Pb loading to the river can now be explained predominantly by natural processes, namely erosion plus chemical weathering.

Among the long list of contemporary environmental contaminants, lead (Pb) is one of the most problematic potentially toxic trace elements because atmospheric contamination by this metal became a global phenomenon^1^,^2^. The geochemical cycle of Pb has been impacted by anthropogenic activities more than any other metal^3^. The use of leaded gasoline around the world from ~1920 to ~1970 was the last but by far the largest episode of the most significant Pb sources to the environment after smelting and refining of Pb and other non-ferrous metals, coal combustion and cement production^4^. Environmental impacts have been studied extensively, and evidence of Pb in both polar regions suggested no area on the earth’s surface has been unaffected by anthropogenic Pb^1^. However, the phase-out and eventual elimination of leaded gasoline in North America and Europe since 1970 and 1980, respectively has precipitated a dramatic decrease in the concentrations of atmospheric Pb^4^,^5^. While the fall in air Pb concentrations has been accompanied by a corresponding drop in human blood Pb^6^, there is no known safe level and blood Pb values are now “seen by society as evidence of its commitment to its own health”^7^.

While declining air Pb concentrations have been documented in environmental archives affected exclusively by atmospheric inputs such as polar snow and ice as well as ombrotrophic (i.e. rain-fed) peat bogs^8^,^9^,^10^ the consequences for surface waters have received less attention. Anthropogenic Pb inputs to global oceans were documented by Clair Patterson^11^ and declines in Pb concentrations in surface seawater subsequent to the phaseout of leaded gasoline have been clearly illustrated for the Atlantic Ocean by Ed Boyle^12^,^13^. In respect to freshwaters, pioneering work on Pb in surface and groundwaters in the 1970’s documented the significant contributions of anthropogenic Pb to aquatic ecosystems at that time^14^. It became clear from these studies that the key to understanding the biological significance of this additional Pb was to distinguish between dissolved and particulate forms: it is the dissolved (<0.45 μm fraction) that is potentially mobile, bioaccessible, bioavailable and toxic^14^. Coincidentally, almost all of the anthropogenic Pb released to the atmosphere from high temperature combustion processes is in the sub-micron fraction^15^. Thus, as far as potential impacts of Pb on human and ecosystem health is concerned, in both air and water, it is the sub-micron fraction which has the greatest relevance. However, studies show that within the conventional dissolved fraction (<0.45 μm) not all the Pb is bioavailable and it can be partitioned into colloidal and mainly ionic (truly dissolved) Pb^16^,^17^,^18^. Ionic Pb is the only fraction that can directly impact the aquatic life^19^. If it were not for the various industrial inputs, almost all of the Pb would be in the particulate fraction, both in the atmosphere^14^ as well as in water^20^, with wind and water erosion, respectively, being the dominant sources of Pb to the surficial environment.

The accurate and precise determination of Pb in the dissolved fraction of natural freshwaters, however, poses several considerable challenges. First, the natural abundance of dissolved Pb in freshwaters is extremely low. Pioneering work from the Patterson lab showed long ago that dissolved Pb in remote streams of the Sierra Nevada watershed is commonly below 15 ng/l^21^. The low concentrations of Pb pose two additional difficulties: 1), the need for tremendous analytical sensitivity, and 2), the grave risks of contamination which are encountered at virtually every stage of the sampling, handling, and analysis process^22^,^23^,^24^,^25^,^26^. While some labs have understood and met these challenges, most have not^27^. In the intervening period there have been many studies of Pb in rivers and lakes^28^,^29^,^30^,^31^,^32^,^33^,^34^,^35^,^36^,^37^,^38^,^39^,^40^,^41^, but there are remarkably few studies providing reliable data for the dissolved fraction. Studies employing the clean lab procedures and protocols developed for polar snow and ice as well as sector-field inductively-coupled plasma mass spectrometer (SF-ICP-MS) have reported Pb values in the dissolved fraction of surface waters and groundwaters in the range of a few ng/l or less^8^,^42^,^43^. Any studies of dissolved Pb in surface waters remote from industrial activity, therefore, need to be able to reliably measure Pb well below the part per trillion (ng/L) concentration range. Studies reveal that the dissolved Pb fraction (<0.45 μm) consists of both colloidal and free (ionic) forms, but it is the ionic Pb fraction that is biologically significant for aquatic organisms^19^. Depending on the relative importance of the colloidal fraction, ionic forms of Pb may be far less abundant than the fraction traditionally defined as “dissolved” i.e., <0.45 μm. The reliable determination of ionic forms of Pb in natural waters, therefore, presents considerable analytical challenges.

To better develop an understanding of the contemporary geochemical cycle of Pb in a major river system and to determine the impact of mining and upgrading activities of the Athabasca bituminous sands on dissolved Pb in the AR, we employ the state-of-the-art metal-free ultra-clean analytical sample collection, handling and analytical procedures developed at the University of Heidelberg for measuring Pb and Pb isotopes in Arctic snow and ice^4^,^44^. These ultra-clean sample collection, handling and analytical procedures have been successfully employed at the SWAMP lab, University of Alberta^45^. We compare total, particulate (>0.45 μm), dissolved (<0.45 μm), colloidal (1 kDa to 0.45 μm) and mainly ionic (<1 kDa) Pb concentrations in the lower reaches of Athabasca River (AR, ~1500 km) in northern Alberta, one of the largest rivers in Canada (http://www.awc-wpac.ca/). The section of the AR selected for study is in the heart of the industrialized region where open pit mines and upgraders of the Athabasca Bituminous Sands (ABS) are found. It was claimed that these commercial activities are a significant source of Pb to air and waters of this region^46^, so samples were collected upstream, downstream, and within the industrialized zone. The main objective of this study is to quantify the impacts of human activities on Pb dissolved in the river. From October 7 to 17, 2014 water samples and suspended solids were collected from thirteen sites along the main stem of the AR, upstream of Fort McMurray and along a transect ~125 km downstream, as well as from five tributary streams and five groundwater sampling locations (Fig. 1). In addition to the water and suspended solids, in March, 2015 snow samples were also collected from peat bogs in the area to characterize Pb in contemporary atmospheric deposition (Fig. S1 and Table S1).

Dissolved Pb concentrations for the main stem and tributaries from this sampling campaign are taken from Shotyk *et al*.^45^, and the rest of the data produced in this study. Sampling, handling and Pb determination in surface and groundwaters are described in detail in the supporting information (SI).

## Lead in the Athabasca River (AR) in perspective

Total Pb in the main stem of the AR (127 ± 58 ng L^−1^; n = 13, Fig. 2A) and its tributaries (119 ± 112 ng L^−1^; n = 5, Fig. 2B) are low: approximately 25 to 55 times below the guideline values established for the protection of aquatic life by the Canadian Council of Ministers of the Environment (CCME)^47^ and ~75% lower than the values for this section of the river reported previously^48^. Total Pb in the AR on average is ~80 times lower than the World Health Organization (WHO) guideline value (10 μg L^−1^) for drinking water. To help put these values into perspective, total Pb in the AR is considerably lower than dissolved Pb in the Nippissing River which was sampled in the most remote section of Algonquin Provincial Park in southern Ontario (dissolved Pb: 306 ± 34 ng L^−1^; n = 3); waters were sampled from this nature reserve (7,653 km^2^) in the autumn of 2006, using the identical sampling and handling protocols described here, but measured in the metal-free, ultraclean lab at the University of Heidelberg in Germany^45^.

**Figure 1:**
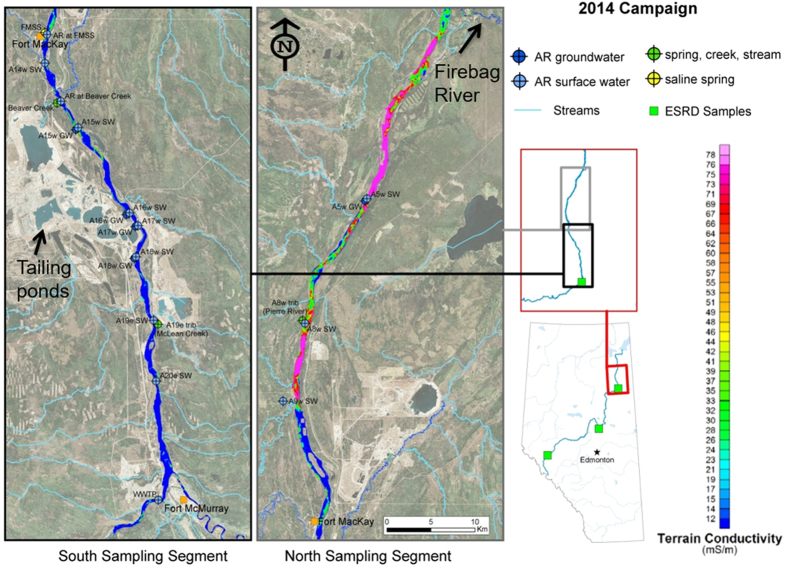
Location of the water samples in relation to the terrain conductivity of the main stem of the Athabasca River (AR). Terrain conductivity data is taken from Gibson *et al*.^71^, and ranges from 12 mS m^−1^ (dark blue) to 76 mS m^−1^ (light pink). The map is created using ArcGIS Desktop (ESRI 2011: Release 10.3. Redlands, CA: Environmental Systems Research Institute, http://www.esri.com/software/arcgis/arcgis-for-desktop) by taking the basemaps and reference layers information through basemap imagery (Source: Esri, DigitalGlobe, GeoEye, i-cubed, USDA, USGS, AEX, Getmapping, Aerogrid, IGN, IGP, swisstopo, and the GIS User Community).

**Figure 2:**
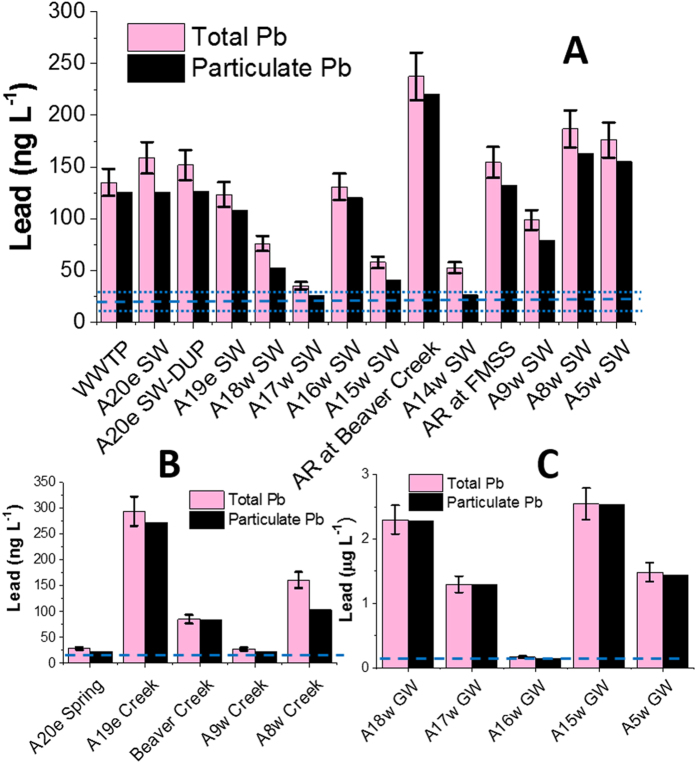
Total and particulate lead (Pb) concentrations in the (**A**) main stem of the Athabasca River, (**B**) tributary streams and (**C**) groundwater. The bars in the graphs show the average Pb concentrations of triplicate samples and error bars are the standard deviations. The dashed lines in the graphs show the average dissolved Pb and dotted lines show the one standard deviation. The dissolved Pb in the main stem AR and tributaries were reported previously (Shotyk *et al*.^45^), but the dissolved Pb in groundwater is generated in this study. The results show that almost all the Pb is in particulate form. Note: while the concentrations of total and dissolved Pb in groundwater presented here represent accurate and precise measurements, the abundance of particulate matter in the groundwater samples is partly a reflection of the procedure employed (drive point wells) to collect them. The values presented here should not be viewed as the final word on the topic, rather the starting point: research on these groundwaters is ongoing.

To determine the possible impact of ABS mining and upgrading activities, total Pb concentrations were compared between the sites located on the AR adjacent to the tailing ponds, mining activities and bitumen upgraders (sites A18 to A9; Fig. 1) and the sites far from industry (A8 and A5; Fig. 1). No significant difference (P = 0.16; two tailed Student’s t.test) was found between the total Pb concentration in the industrial zone (105 ± 67 ng L^−1^; n = 8) and in the area far from these activities (181 ± 8 ng L^−1^; n = 2). Total Pb in the main stem of the AR (127 ± 58 ng L^−1^; n = 13, Fig. 2A) and the five tributary streams (119 ± 112 ng L^−1^; n = 5, Fig. 2B) show no significant differences (P = 0.85; two tailed Student’s t.test). Although it has been suggested that there are significant inputs of Pb to the AR from the industrial development of the bituminous sands^46^, there is no evidence of this in the total Pb concentrations which is the fraction upon which the CCME guideline for the protection of aquatic life is based. Greater concentrations of total Pb were found in the groundwater samples (0.2 to 2.5 μg L^−1^; Fig. 2C), but this is a reflection of the greater abundance of particles in groundwater compared to the surface waters (Figs S2 and S3).

To distinguish natural from anthropogenic Pb in atmospheric aerosols and other environmental monitoring media such as snow, ice, moss and peat, Pb can be normalized to a conservative lithophile element such as thorium (Th) or scandium (Sc)^10^,^49^,^50^. The normalization provides an indication of the extent to which industrial activities have contaminated the sample, relative to the corresponding natural background (crustal) ratio in soil-derived dust particles. The same approach can be used with bulk water samples, to account for the contribution of suspended mineral matter to total concentrations. The ratio of Pb to Th in the bulk surface water samples from the main stem of the AR (2.5 ± 0.3; n = 13) are similar to those of groundwater (2.2 ± 0.3); these are remarkably similar to the Pb to Th ratio in the Wye River (2.4 ± 0.7; n = 3), a small farm stream sampled near Elmvale, Ontario^43^. The total Pb concentration in the Wye River (74 ± 5 ng L^−1^; n = 3) is lower than in the AR, but this most likely reflects the differences in flow rates: 623 m^3^ sec^−1^ on average, in Fort McMurray compared to ~1 m^3^ sec^−1^ in the Wye River (Environment Canada). Simple Pearson Correlation between total Pb and total Th in the surface water of the AR main stem, its tributaries and groundwater (*r* = 0.93, 0.98 and 0.94, respectively) reveals that overall the Pb distribution in water follows Th remarkably well, which suggests that Pb concentration in the river are almost exclusively a reflection of the abundance of mineral particles.

## Particulate (>0.45 μm), dissolved (<0.45 μm), colloidal (1 kDa to 0.45 μm) and mainly ionic Pb (<1 kDa)

Dissolved Pb is very low in both the main stem of the AR (20 ± 7 ng L^−1^: n = 13, Fig. 2A) as well as the tributary streams (25 ± 30 ng L^−1^; n = 5, Fig. 2B). Based on these values and the total concentrations presented here, it is clear that almost all of the Pb in the AR is associated with particulate fraction (Fig. 2A,B). Over 90% of the total Pb measured in the Wye river water was also found in the particulate form (data not shown). Here we also report the first reliable measurements of dissolved Pb (123 ± 82 ng L^−1^; n = 5) in the groundwaters which feed the AR (Fig. 2C): even in the groundwaters, almost all of the Pb is in the particulate fraction.

**Figure 3:**
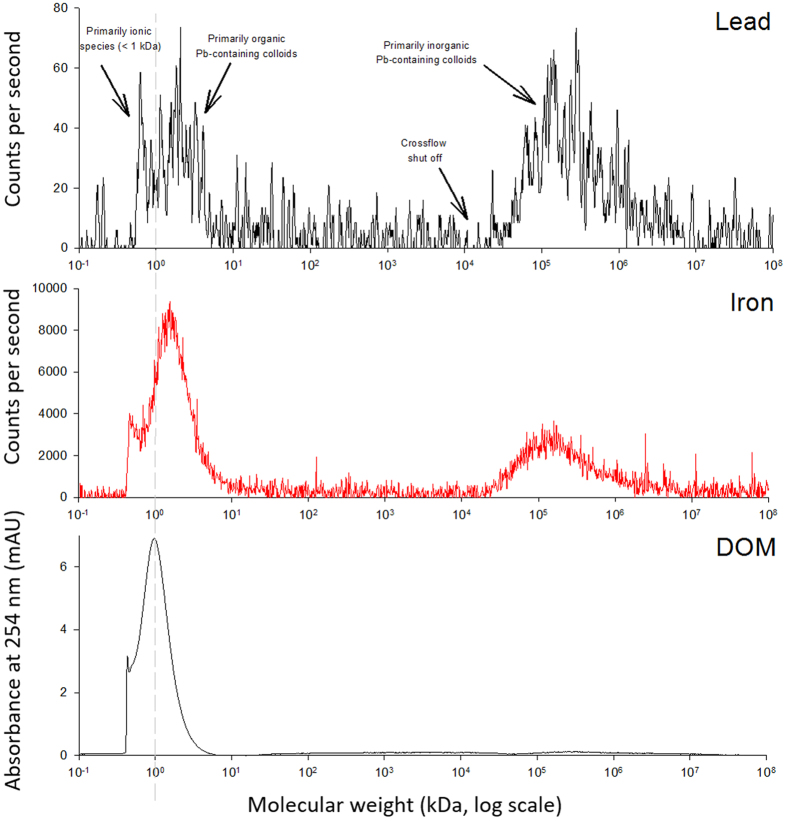
Molecular weight distributions of dissolved (<0.45 μm) lead (Pb), iron (Fe) and organic matter (DOM) in the Athabasca River (AR) surface water sample. DOM and Fe peaks at ~0.98 and 1.7 kDa, and peak at >20 kDa (middle panel) show the primarily organic and primarily inorganic colloids, respectively. Fractionation of dissolved Pb (upper panel) shows Pb association with both organic and inorganic colloids. Data is baseline corrected and Pb data is smoothed (5 point running average). Here we define colloidal Pb = Pb associated with organic colloids + Pb associated with inorganic colloids, and mainly ionic Pb = Dissolved Pb - Colloidal Pb. The crossflow was shut off to release large material at a retention time corresponding to a molecular weight of ~104. Thus, the specific molecular weight of material that is larger than this cannot be determined.

**Figure 4:**
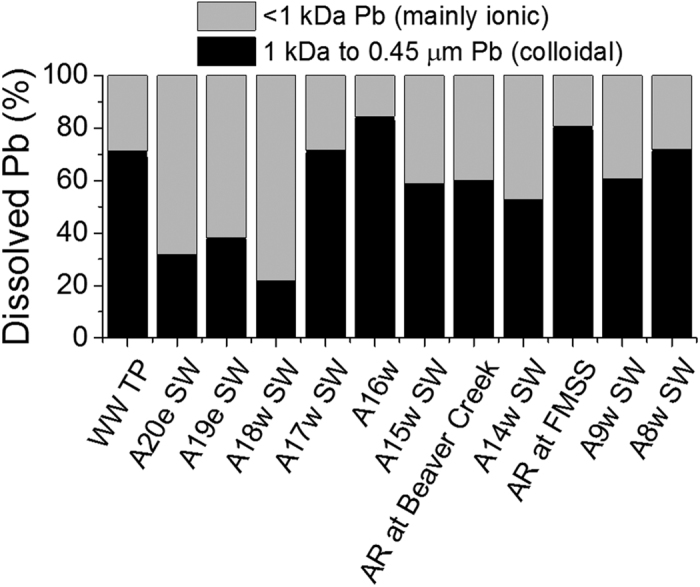
Distribution of dissolved lead (<0.45 μm fraction) into colloidal (1 kDa to 0.45 μm) and mainly ionic species (<1 kDa).

Dissolved Pb in the AR is very low and fractionating the dissolved Pb into colloidal (1 kDa to 0.45 μm) and mainly ionic (<1 kDa) forms reveals that on average ~60% of the dissolved Pb (~20 ng L^−1^) is bound with organic and inorganic colloids which means that the bioaccessible Pb concentration is on the order of 8 ng L^−1^ (Figs 3 and 4). This is similar to the proportion of colloidal Pb measured in an estuary using crossflow ultrafiltration (64 ± 9%; Wen *et al*.^51^, and to the proportion measured in stream water during snowmelt using AF4-ICP-MS with a 1 kDa membrane (59%; Stolpe *et al*.^52^). Curiously, Neubauer *et al*.^53^ found that 116 and 70% of dissolved (<0.2 *μ*m) Pb was colloidal in two peat bog drainage samples using AF4-ICP-MS with the 0.3 kDa membrane, whereas 89 and 96% was respectively found to be colloidal in the same samples using a stirred ultrafiltration cell. Comparing results across studies is challenging given disparities in analytical methods, DOM sources, physicochemical conditions, and filter pore sizes. The distribution of Pb amongst different size fractions also depends upon its source (e.g. wind erosion of soils vs high temperature combustion vs reprocessed colloids), so that the useful comparison of size distributions across studies requires more thorough analysis of trace element properties.

## Characterizing particulate Pb

**Figure 5:**
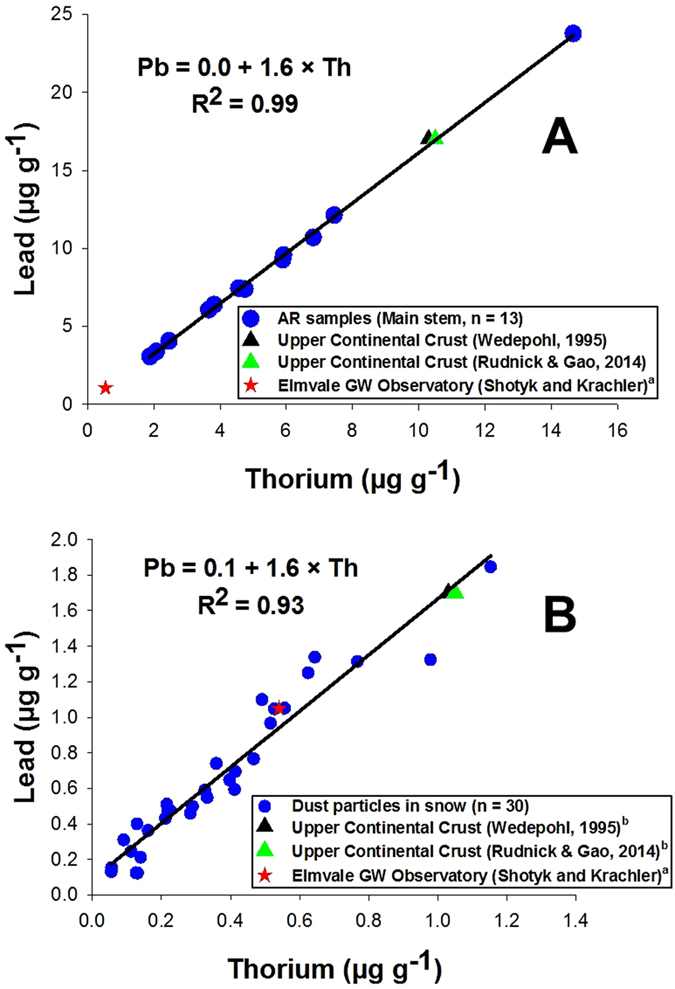
Scatter plots between lead (Pb) and thorium (Th) concentrations in (**A**) the suspended solids in the main stem of the Athabasca River (AR) and (**B**) the dust particles in snow samples collected from five peat bogs in the study area. The slopes of the regression lines (1.6) between Pb and Th in both graphs are similar to the ratio in the Upper Continental Crust^57^,^58^. ^a^The Pb/Th ratio in the water samples studied here is also compared with the Pb/Th ratio in groundwaters collected from the HDPE well of the Elmvale Groundwater Observatory. The HDPE tubes and screen used to construct this well (known as EGO-2) are from Rotek (Denmark); they had been shipped to the University of Heidelberg, Germany, for cleaning in HNO_3_. After rinsing, drying in metal-free, laminar flow clean air cabinets, and packing in sealed PE bags, the well materials were shipped from Germany to Canada and installed near (3 m) but hydrologically upgradient of EGO-1, a flowing, artesian groundwater sampling well constructed entirely of stainless steel. Water samples are collected from the EGO-2 (HDPE) well via a purpose-built, acid-cleaned Teflon valve, within a metal-free, laminar flow clean air cabinet. Both of these flowing, artesian wells near the Village of Elmvale, Ontario, emanate from an aquifer at a depth of 13 m: together they are known as the Elmvale Groundwater Observatory and are dedicated to the study of trace metals in water. The water samples were collected in March of 2011 and all measurements performed at the University of Heidelberg, Germany using an Element 2 ICP-SMS (Shotyk and Krachler, unpublished data). The data presented here from EGO-2 represents water samples (n = 10) that were collected using acid-cleaned HDPE bottles provided by Dr. Jiancheng (James) Zheng of the Geological Survey of Canada, Natural Resources Canada, Ottawa. In fact, these are the same types of bottles, prepared in the same manner, used in the study of Pb in the ice core from Devon Island, Nunavut, in the Canadian Arctic^4^,^44^. The Pb/Th ratio in the particulate and dissolved fractions of the AR is remarkably similar to the Pb/Th ratio found in this groundwater. ^b^Pb/Th ratio in the Upper Continental Crust/10.

We developed a precise mechanical procedure to recover each polytetrafluoroethylene (PTFE) filter membrane from its plastic housing (Fig. S4) along with an effective protocol to digest the particles recovered (SI). The Pb concentrations in these suspended solids (Figs S5A and B), whether they were recovered from the main stem of the AR (8.6 ± 5.6 μg g^−1^, n = 13) or the tributary streams (4.7 ± 2.8 μg g^−1^, n = 5), are similar to the values for sedimentary rocks^20^ which constitute the Western Canadian Sedimentary Basin^54^ and are similar to the average Pb content of sandstones and limestones (7 μg g^−1^ Pb)^55^. These Pb values in the suspended solids in the AR are similar to the Pb concentrations in the suspended particles (2.5 μg g^−1^) reported in Siberian rivers draining basalts^56^. Among the different samples, Pb concentrations in the suspended solids at site AR at Beaver Creek (~24 μg g^−1^ Pb) was about 3 times higher than the average Pb in the other sites. Comparing the TSS results showed that this site (AR at Beaver Creek) contained more TSS (0.7 g/L) than the average TSS (0.3 g/L) at other sites. The groundwater samples have slightly greater Pb concentrations in the particulate fraction, (15.1 ± 3.1 μg g^−1^, n = 5; Fig. S5C), but these still resemble the abundance of Pb in the Upper Continental Crust which is 17 μg g^−1^ according to both Wedepohl^57^ and Rudnick and Gao^58^. We suspect that the difference in Pb concentrations of the particulate fraction in groundwater versus surface water may be a particle size effect. A linear regression of Pb against Th in the suspended solids of the AR (Fig. 5A) shows a remarkable correlation ([Pb] = 1.6 × [Th] + 0.0; R^2^ = 0.99). The slope of the regression line is identical to the Pb/Th ratio (1.6) for the Upper Continental Crust^57^,^58^. Taken together, the results show that Pb in the AR is almost exclusively in the form of mineral particles derived from physical weathering and erosion within in the watershed, with a tiny contribution to the Pb inventory from the dissolved fraction which must be supplied by chemical weathering. In contrast to the AR, the average Pb concentration reported in the suspended solids of world rivers (25–100 μg g^−1^) is far higher^59^,^60^,^61^.

## Lead in snow

**Figure 6:**
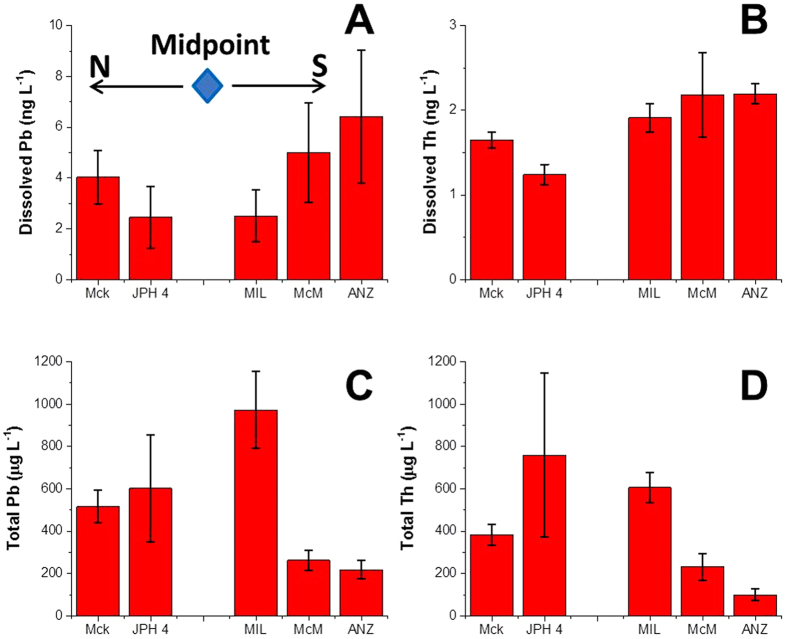
Concentrations of (**A**) dissolved (<0.45 μm) lead, (**B**) dissolved thorium, (**C**) total lead and (**D**) total Th in snow samples collected from five peat bogs in the study area, the locations of bogs are shown in Fig. S1. The bars in the graph show average Pb or Th concentrations of triplicate samples collected from every site and the error bars represent one standard deviation.

Snow pack sampling has been used to suggest that atmospheric deposition is an important source of industrial Pb to the AR^46^. In March 2015, we collected snow samples from peat bogs in several areas close to the ABS mining and upgrading activities, and measured total as well as dissolved (<0.45 μm) Pb (Fig. S1 and Table S1). The details of the sample locations, snow sampling, handling and Pb determinations are given in the Supporting Information. The average concentration of dissolved (<0.45 μm) Pb in the snow samples was 4.2 ± 0.7 ng L^−1^ (n = 5; Fig. 6A). The dissolved Pb concentrations in both the snow samples and the AR main stem surface water are low, furthermore, the dissolved Pb in the snow collected from the peat bogs closest to the industrial activity is ten times lower than the average dissolved Pb in the AR main stem surface water. To put the dissolved Pb concentration in the snow into perspective, Pb concentrations in contemporary snow (1994 to 2004) from the Arctic are ten times greater^4^. In fact, the average concentration of Pb from the “cleanest” section of the ice core collected on Devon Island, Nunavut, representing snow accumulation from ca. 5,000 to 8,000 years ago, is 5.1 ± 1.4 ng/L^44^. Dissolved Pb in the snow samples follows the distribution of dissolved Th (n = 5; Fig. 6B). Average total Pb concentrations in the snow was 749 ± 421 μg L^−1^ (n = 5; Fig. 6C) compared with total Th (347 ± 294 ng L^−1^, n = 5; Fig. 6D); Pb showed a significant positive correlation (*r* = 0.99, *P* < 0.01) with total Th. Moreover, the ratio of total/dissolved Pb in the snow (180) matches remarkably well the total/dissolved Th (189). These results show that dust particles (primarily mineral matter) in the snow are the main factor controlling Pb concentrations. Comparing the Pb and Th concentrations in the dust particles in snow, a significant positive correlation was found ([Pb] = 1.6 × [Th] + 0.1; R^2^ = 0.93, Fig. 5B). Similar to the suspended solids in the AR, the slope of the regression line is identical to the Pb/Th ratio (1.6) for the Upper Continental Crust^57^,^58^. If mining and upgrading of ABS was a significant source of Pb to the air, the Pb concentrations in the snow from this region should be enriched, relative to the abundance of Th in snow. Clearly, that is not the case and the Pb/Th ratios fail to identify a detectable anthropogenic contribution.

## Ecological significance of Pb in the AR?

The Peace-Athabasca Delta (PAD) is one of the largest freshwater deltas in the world, and of tremendous ecological significance. There are legitimate concerns about a range of environmental impacts on the delta, including contaminants received from the AR watershed^62^, one of them being Pb. It was suggested long ago^14^,^59^ that more than 90% of the Pb in rivers should be in the particulate fraction. Although many studies have found that Pb in most rivers is dominated by the particulate fraction^63^,^64^,^65^,^66^, in the past there had been a large anthropogenic contribution which apparently dominated the dissolved fraction and contributed in a significant way to total Pb concentrations. As anthropogenic emissions of Pb to the atmosphere have declined worldwide, it has become increasingly difficult to measure dissolved Pb in natural waters. The lack of reliable measurements of Pb in the dissolved fraction has limited our understanding of the potential ecological significance of Pb in aquatic systems: this fraction is the key to bioaccessibility and bioavailability of Pb. In the AR, using the metal-free ultraclean procedures and protocols developed for polar snow and ice, we see that Pb in the dissolved fraction is almost inconsequential. Further fractionation of dissolved Pb into colloidal (1 kDa to 0.45 μm) and mainly ionic (<1 kDa) forms reveals that a significant proportion (~60%) of the traditionally defined as “dissolved” Pb fraction is, in fact, bound with colloids, and these are expected to have limited bioavailability.

To bring these results into a broader prospective and understand the overall Pb inputs to the PAD, based on dissolved Pb reported previously (Shotyk *et al*.^45^), and Pb in the suspended solids presented here, we find that almost the entire Pb load to the PAD is in the particulate fraction, in the form of mineral particles, whereas dissolved Pb accounts for no more than 1–2% of total Pb. Moreover, within the “dissolved” fraction, approximately 60% of the Pb is bound with colloidal particles (Fig. 4). Again, with the hope of bringing perspective to the data, consider that air Pb emissions from all industrial sources in Alberta was 1757 kg in 2014^67^. The AR watershed (150,000 km^2^) represents approximately 23% of the surface area of the Province of Alberta. If 23% of all annual industrial Pb emissions in Alberta were to become dissolved in the river, it would only increase the concentration of dissolved Pb i.e., the <0.45 μm fraction (20 ng L^−1^) by a factor of ~2. If the same mass of industrial Pb was exclusively in the form of particulates, it would contribute less than 2% to the Pb inventory in the suspended solids.

## Summary

International efforts over decades to reduce anthropogenic emissions of Pb to the environment have successfully reduced air Pb concentrations worldwide^6^, and benefits to aquatic ecosystems such as the AR are now becoming apparent as well. Lead in the AR is clearly dominated by natural inputs, mainly erosion, to such an extent that an anthropogenic component is now difficult to discern.

## Experimental

From October 7 to 17, 2014 using acid-cleaned polypropylene (PP) bottles, raw surface water samples were collected from thirteen sites along the main stem Athabasca River (AR) starting upstream of Fort McMurray and travelling downstream a distance of approximately 125 km. The physicochemical properties of the main stem AR water samples are provided in Table 1. Along this route, water was also collected from five tributary streams draining into the AR and groundwater from five sites (Fig. 1). The sampling sites were selected to represent the upstream locations (WWTP, A20 and A19 sites), the industrial zone (from site A18 to A9 on the river, sites that are adjacent to tailing ponds and bitumen upgraders) as well as the sites far from industry (A8 and A5; Fig. 1). All the sample collection, handling and measurements were carefully performed employing the protocols and procedures developed at the University of Heidelberg, Germany for the determination of Pb and stable Pb isotopes in Arctic ice cores^4^,^44^. The raw water samples (2 mL) were digested with 5 mL of double distilled concentrated nitric acid (HNO_3_) using high pressure microwave digestion (Ultraclave, MLS Leutkirch, Germany) and Pb determined using inductively-coupled plasma mass spectrometry (ICP-MS; iCAP Qc). The details of the determination of Pb in the water samples are provided in the Supporting Information (SI).

Particulate Pb in the main stem AR, tributary streams, and groundwater samples was calculated by subtracting the dissolved Pb from total Pb:


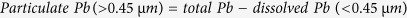


Dissolved Pb concentrations for the main stem and tributaries from this sampling campaign have been presented elsewhere (Shotyk *et al*.^45^). Dissolved Pb in the groundwater samples was measured in this study.

The distribution of Pb into colloidal (1 kDa to 0.45 μm) and mainly ionic (<1 kDa) forms within the dissolved fraction (<0.45 μm) was determined using asymmetrical flow field-flow fractionation (AF4) equipped with an auto injector (AF2000 MF and PN5300, respectively, Postnova Analytics, Salt Lake City, Utah, USA), coupled to a UV-Visible absorbance detector (G4212 DAD, Agilent Technologies, Santa Clara, California, USA) and ICP-MS (iCAP Qc, Thermo Fisher). The use of the term “mainly ionic Pb” assumes that Pb in this size fraction is mainly in an ionic form such as Pb^2+^, an inorganic complex such as PbCl^+^ or PbSO_4_^0^, a low molecular weight organic complex, or some combination of these. The fractionation program and peak deconvolution method were adapted from earlier work^68^,^69^, and are discussed in detail in the SI.


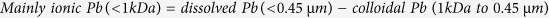



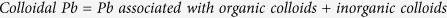


In the field, water samples for dissolved Pb were filtered through acid-cleaned 0.45 μm PTFE filters into bottles containing HNO_3_. The filters containing the suspended particles (>0.45 μm) were stored, and preserved for later study. An excellent mechanical setup was devised to cut and open the filter housing to acquire the filter membranes without any loss of suspended sediment (Fig. S4). The suspended sediments were digested in double distilled concentrated HNO_3_ (6 mL) and HBF_4_ (0.2 mL) using high pressure microwave digestion (Ultraclave, MLS) and Pb determined using ICP-MS (iCAP Qc). The details of the sample handling, preparation and measurement is provided in the SI. The micromorphology of the suspended solids were study using scanning electron microscope following Javed *et al*.^70^, the details of the method are provided in SI.

In addition to water and suspended solids, snow samples were also collected from the Athabasca Bituminous Sands (ABS) mining and upgrading area (Fig. S1). The details of the snow sampling, handling and measurements for dissolved and total Pb and Th concentrations, and Pb and Th concentrations in the dust particles in snow are provided in the SI. Briefly, snow samples collected in acid cleaned PP bottles (1 L) were thawed in the metal free, laminar flow clean air cabinets in the SWAMP laboratory, filtered through acid cleaned 0.45 μm PTFE filters and dissolved Pb and Th concentrations determined using ICP-MS (iCAP Qc).

## Additional Information

**How to cite this article:** Javed, M. B. *et al*. Size-resolved Pb distribution in the Athabasca River shows snowmelt in the bituminous sands region an insignificant source of dissolved Pb. *Sci. Rep.* **7**, 43622; doi: 10.1038/srep43622 (2017).

**Publisher's note:** Springer Nature remains neutral with regard to jurisdictional claims in published maps and institutional affiliations.

## Supplementary Material

Supplementary Information

## Figures and Tables

**Table 1:** Physicochemical properties of the main stem AR water samples.

Sample ID	Water Temp. °C	pH - lab	Conductivity - lab μS cm^−1^	Dissolved Oxygen mg L^−1^	DOC^a^ C mg L^−1^	Alkalinity mg L^−1^	TSS^b^ mg L^−1^
WWTP	7	8.3	324	13	7	124	232
A20e-SW	8	8.1	279	13	8	93	217
A20e-SW-DUP	7	8.0	272	13	8	91	—
A19e-SW	7	8.2	262	13	6	105	193
A18W-SW	7	8.2	301	13	6	100	210
A17W-SW	7	8.2	239	13	6	116	207
A16W-SW	7	8.2	338	12	6	113	224
A15W-SW	8	8.2	298	12	6	108	215
River at Beaver Creek	8	8.2	329	12	7	102	212
A14W-SW	8	8.2	266	12	7	106	222
River at FMSS	8	8.2	274	13	7	102	216
A9W-SW	8	8.1	305	12	8	103	207
A8W-SW	8	8.2	338	12	8	104	212
A5W-SW	8	8.2	317	12	8	161	200

^a^Dissolved organic carbon. ^b^Total suspended solids.
