# Supplementary material for: Size-resolved Pb distribution in the Athabasca River shows snowmelt in the bituminous sands region an insignificant source of dissolved Pb
Source: Sci Rep. 2017 Mar 6;7:43622. doi: 10.1038/srep43622 (PMC5338012; doi:10.1038/srep43622)
Supplement: Supplementary Information [file srep43622-s1.pdf]

Supporting Information

Size-resolved Pb distribution in the Athabasca River shows snowmelt in the bituminous sands region an insignificant source of dissolved Pb

Muhammad Babar Javed, Chad W. Cuss, Iain Grant-Weaver, William Shotyk*

Department of Renewable Resources, University of Alberta, AB CANADA T6G 2H1

* Correspondence to: W. Shotyk (shotyk@ualberta.ca)

Sampling of surface water and suspended solids (>0.45 µm) sampling

AR main stem and tributary streams

From October 7 to 17, 2014 using acid-cleaned polypropylene (PP) bottles, raw surface water samples were collected from thirteen sites along the main stem Athabasca River (AR) starting upstream of Fort McMurray and travelling downstream a distance of approximately 125 km (Fig. 1).

All the surface waters were collected using the 'clean hands/dirty hands' sampling method, where two persons were fully involved to obtain the samples. The details of the sampling method are provided previously (Shotyk et al. [45]). Briefly, surface water sampling from the main stem AR was performed using a small boat anchored ~80 m from the shore. The water samples were collected using an acid-cleaned 125 mL PP transfer bottle opened and capped approximately 30 cm below the water surface. The raw water samples (without any filtration or acidification) for total Pb concentrations were collected from the transfer bottles into acid cleaned 125 mL PP bottles. The raw surface water samples were also collected from 5 tributary streams draining into the AR for total Pb concentrations following the sampling procedure described above. The waters collected from streams were taken from the central portion of the flow and always taken upstream of the person doing the sampling.

For a separate study of the dissolved trace metals in the AR (Shotyk et al. [45]), all the water samples from main stem of the river and its tributary streams were collected from the transfer bottle using an acid-cleaned PP syringe, and then filtered in the field using acid-cleaned 0.45 µm PTFE filters into acid-cleaned 125 mL PP bottles containing double-distilled nitric acid (HNO_3, 2 µL/mL) to preserve the metals. The PTFE filters used in the field were saved and later used to determine the Pb contents in the suspended solids.

After sample collection, each water sample and filter was separately double bagged in polyethylene (PE) ZipLock bags and kept inside a cool box in the field until being refrigerated (4°C) at the end of the day. Basic water quality parameters such as temperature, conductivity, oxidation-reduction potential, dissolved oxygen and pH were measured at every sampling site using a portable multimeter (YSI Pro Plus, Yellow Springs, OH, USA). A summary of the basic water quality parameters was given previously (SI Table 1 and 2, Shotyk et al. [45]), along with the GPS coordinates of the sampling location.

Groundwater sampling

Groundwater samples were collected from five sites, namely A5, A15, A16, A17 and A18 (Fig. 1) along the AR using stainless steel drive point wells as shown in Fig. S2. The wells were cleaned with detergent and repeatedly rinsed with deionized water in the ultraclean metal-free SWAMP laboratory at the University of Alberta. After cleaning, the wells were dried in the clean air cabinets (CAC) and packed in PE bags before being brought into the field. To collect the groundwater samples, the drive point wells were installed at appropriate depths (~90 to ~170 cm depending on the depth of the aquifer). After installation, an acid clean PP tube was inserted into the well to remove groundwater using a peristaltic pump (Fig. S2). The wells were purged at least 3 times of the volume of the well. The conductivity of the water obtained from the well repeatedly measured, and once the conductivity stabilized, the first groundwater samples were collected. To collect the samples, water from the acid-cleaned PP tube was collected into an acid cleaned 125 mL PP transfer bottle. For total Pb concentrations, raw water samples were collected into acid-cleaned 125 mL PP bottles. Groundwater samples for the dissolved trace metals were collected from the transfer bottle using an acid-cleaned syringe, and then filtered in the field

using acid-cleaned 0.45 µm filters into acid-cleaned 125 mL PP bottles containing double-distilled nitric acid (HNO_3, 2 µL/mL) to preserve the metals. The PTFE filters were also saved to allow Pb to be determined in the suspended solids.

Total lead

To determine total Pb in the main stem AR, tributary streams and groundwater samples, approximately 2 mL of the raw water samples were digested in double distilled nitric acid (dd HNO_3, 3 mL) using a high-pressure microwave digestion unit pressurized to 50 bar using argon (Ar) following a multistep digestion procedure[50].

Lead concentrations in the digested solutions were determined using the quadrupole inductively-coupled plasma mass spectrometer (ICP-MS; iCAP Qc, Thermo Fisher Scientific, Waltham MA, USA) housed in the metal-free ultraclean SWAMP laboratory at the University of Alberta. Samples were analyzed in Kinetic Energy Discrimination mode (KED), with helium (He) as the collision gas to eliminate the polyatomic interference during analysis, with a 0.03 s dwell time and 30 sweeps: Pb concentrations were obtained as the average of the three main runs during data acquisition. In addition to Pb, thorium (Th) was also determined in all the samples using ICP-MS. Standard stock solutions purchased from Spex CertiPrep (Metuchen, NJ, USA) were used to create the calibration curves for Pb and Th. A linear regression was obtained for both the elements before measuring the samples. To account for any instrument drift during analysis, indium (In) was used as an internal standard.

Quality control

For quality assurance and quality control (QA/QC), certified reference materials purchased from the National Institute of Standards and Technology (NIST 1640a) and LGC

standards, USA (SPS-SW2) were analyzed along with the samples to assess instrument calibration and performance. The LOD and LOQ for both Pb and Th, along with the accuracy and precision of the measurements (obtained from the analysis of reference materials), is provided in Table S2.

In addition to the reference materials, lab blanks and field blanks were prepared in triplicate as follows: bottles were filled with MilliQ water and acidified (2 µL/mL) using dd HNO_3. Field blanks were brought with the sampling team into the field each day, while lab blanks remained refrigerated in the SWAMP Lab. Upon analysis, all the lab and field blanks were below the instrument LOD for both Pb and Th.

Particulate (> 0.45 µm), colloidal (1 kDa to 0.45 µm) and mainly ionic lead (<1 kDa)

To calculate the particulate Pb concentrations in the main stem AR, tributary streams and groundwater, dissolved Pb (< 0.45 µm) concentrations were subtracted from the total Pb concentrations. Dissolved Pb concentrations in the main stem AR and tributaries were taken from the previous study (Shotyk et al.[45]), and for groundwater samples the dissolved Pb was determined in this study.

The distribution of Pb in the colloidal and mainly ionic forms within the dissolved fraction (< 0.45 µm) was determined using asymmetrical flow field-flow fractionation (AF4) equipped with an auto injector (AF2000 and PN5300, respectively, Postnova Analytics, Salt Lake City, Utah, USA), coupled to a UV-Visible absorbance detector (G4212 DAD, Agilent Technologies, Santa Clara, California, USA) and ICP-MS (iCAP Qc) following the method described by Cuss et al (in preparation).

After filtration the water through acid-cleaned 0.45-μm syringe filters, the samples were collected in polypropylene (PP) bottles that were acid-cleaned in a certified class 10000 clean room using in-house dd $HNO_{3,}$ but no acid was added to the bottles. The AF4-ICP-MS system was housed in a certified class 1000 clean room, and all reagents and standards were prepared using ultrapure Milli-Q water (MQW; ≥ *18.2* MΩ·cm at 25 °C), in class 100 laminar flow cabinets equipped with HEPA filters. The AF4 system and all the tubing were cleaned to assure extremely low background by circulating 2% dd HNO_3 at a flow rate of 0.1 mL min^{-1} for two weeks. For the eluent reservoir and autosampler vials only acid-cleaned PP containers (as opposed to glass) were used to minimize Pb leaching.

The AF4 fractionation procedure was adapted from an earlier method using a 300-Da polyethersulfone membrane with a 500-μm PTFE spacer[68], and optimized for the separation of dissolved trace-metal species. Briefly, the carrier fluid buffer was made using ultrapure (> 99.999%) ammonium carbonate (Sigma-Aldrich, St. Louis, Missouri, USA) adjusted to pH 8.3 and a conductivity of 300 μS cm^{-1} using Milli-Q water and trace-grade hydrochloric acid (Thermo Fisher Scientific)[54]. Flow settings were as follows: injection flow, 0.2 mL min^{-1}, crossflow 2.1 mL min^{-1}, channel flow 0.7 mL min^{-1}. Focussing and elution were respectively conducted for 6 and 23 minutes, whereupon the crossflow was linearly decreased to zero over a one-minute period and elution was continued for 20 additional minutes. Each fractionation was concluded with a five-minute cleaning step (4 mL min^{-1} with the purge valve open), and blanks were analyzed between samples to eliminate memory effects. Size calibrations were conducted twice daily using a mixture of bromophenol blue (0.69 Da) (Sigma-Aldrich) and four polystyrene-sulfonate sodium salt size standards with molecular weights of 0.89, 3.42, 10.2, and 20.7 kDa (PSS-Polymer Standards Service-USA, Inc., Amherst, Massachusetts). The absorbance

at a wavelength of 254 nm was measured as a proxy for DOM concentration. Each sample and calibration analysis run was concluded with a 5-minute cleaning step (tip flow 4 mL min-1 with purge valve open). A complete blank run (including an additional 5 min. of cleaning) was analyzed after every sample and calibration run. Negligible carryover of DOM or calibration standards (A254 peak height < 0.1 mAU compared to ~ 10 mAU for samples) was observed in the blank analyses.

A high-pressure injection valve (Model 9725, Rheodyne , California, USA) equipped with a 0.3-mL sample loop (same size as auto injector) was installed downstream of the UV-Vis detector to calibrate the ICP-MS and measure the concentration of the whole sample under identical flow conditions. To acidify the sample and dissolve particles prior to entering the ICPMS, a solution of 10% in-house double-distilled trace-grade HNO_3 (Thermo Fisher Scientific) was introduced at a flow rate of 0.1 mL min^{-1} through a micro-mixing tee (IDEX, Lake Forest, Illinois, USA) downstream of the injection valve. Calibrations were conducted daily by injecting a multi-element standard (Solution 2A, Spex CertiPrep, Metuchen, New Jersey, USA), diluted to concentrations of 0.01, 0.1, 1, and 10 ppb. Calibration and sample concentrations were determined by integrating peak areas using Thermo Fisher software (Qtegra). Since no integrable areas were measured in the blanks, LODs were determined using the standard error and sensitivity of the calibration curve (Currie, 1999 and Neubauer et al., 2013).

The areas of the void, organic matter-associated and oxyhydroxide-associated peaks were determined using statistical deconvolution[69]. Briefly, peaks were deconvoluted by fitting each fractogram for one to three normally-distributed peaks, using three optimum fits selected for each number of peaks. In turn, each optimum fit was selected from 1000 individual fittings from

random starting positions, conducted by optimizing the peak location, height, and area using a modified simplex algorithm. The individual fitting with the lowest root-mean-square error (RMSE) was selected as the optimum fit. From the three optimum fits for each peak number, the change in average RMSE with an increasing number of peaks, average standard deviation in peak location, and visual inspection of fit quality were used as selection criteria for the number of peaks. The amount of unbound Pb was calculated as the total concentration minus the amount associated with organic matter or iron oxyhydroxides, as determined from the deconvoluted peak areas (Fig. 3).

Lead concentration in the suspended solids

To allow Pb concentrations to be determined in the suspended solids (> 0.45 µm) retained on the filter membranes housed in the 0.45 micron filter discs, the filter discs were cut and opened to acquire the membrane containing the sediments. A robust mechanical setup was designed to precisely cut the filter discs to completely recover the filter membrane avoiding any particle loss and to minimize the risk of contamination (Fig. S4). Before cutting, the filters were vacuum-dried to make sure all water was removed and that the particles remained attached to the membrane. After recovery, the membranes containing the solids were digested in dd HNO_3 (6 mL) and HBF_4 (0.2 mL) using the high-pressure microwave following the same program used for raw water digestion[50].

For QA/QC, two SRMs (IAEA/SL-1 and IAEA/Soil7, International Atomic Energy Agency, Vienna) were digested. The Pb and Th concentrations were determined using the iCAP Qc's (KED mode) as described above. The SRMs yielded excellent recoveries for both elements (Table S3).

Scanning electron microscopy (SEM) of suspended solids (> 0.45 μm) in the AR

To study the micromorphology of the suspended solids on the filters, a high spatial resolution (~3 nm) JEOL scanning electron microscope (SEM; Field Emission) was used in the Department of Earth Sciences at the University of Alberta. The details of the SEM procedure are provided elsewhere[70]. Briefly, the SEM was used to obtain high-resolution digital images of surface textures and microstructures at high magnifications (20 to 250,000x) with a resolution of approximately ~3 nm. The filter membranes containing the suspended sediments (> 0.45 μm) were mounted on an aluminum stub using a double-sided carbon tape and examined using the SEM under variable pressure (VP) mode without any pre-treatment or coating of the particles.

Snow sampling

Snow samples were collected in March, 2015 from five peat bogs, namely McK, JPH 4, MIL, McM and ANZ from the study area (Fig. S1 and Table S1). These bogs are being used to reconstruct atmospheric metal deposition using *Sphagnum* moss for contemporary inputs[50] and peat for retrospective analysis[9]. The sampling sites were selected based on the distance from the midpoint of the mining and upgrading activities as shown in Fig. S1 and Table S1. Snow samples were collected into acid-cleaned wide mouth 1L PP bottles. After collecting the snow, the bottles were double packed in PE ZipLock bags and kept frozen during transportation to the laboratory.

In the metal-free ultraclean SWAMP laboratory at the University of Alberta, the snow samples were thawed in metal free, laminar flow clean air cabinets. After melting, the samples were filtered through acid-cleaned 0.45 μm PTFE filters using acid-cleaned PP syringes. After filtration, the samples were acidified to 2% HNO_3 using dd HNO_3 and dissolved Pb and Th

concentrations were determined using ICP-MS. For total concentrations of Pb and Th, ~2 mL of the melted snow samples were digested in dd HNO_3 (3 mL) and HBF_4 (0.1 mL) using a high-pressure microwave digestion unit pressurized to 50 bar using argon (Ar) following a multistep digestion procedure[50]. To determine the Pb and Th concentrations in the dust particles in snow, the dust particles were extracted from the snow and digested in dd HNO_3 (6 mL) and HBF_4 (0.2 mL) using the high-pressure microwave following the same program used for raw water digestion[50]. Lead and Th concentrations in the digested solutions were determined using ICP-MS.

References

Currie, L. A. Detection and quantification limits: origins and historical overview. *Anal. Chim. Acta.* **391**, 127-134 (1999).

Cuss, C.W., Grant-Weaver, I., Shotyk, W. Routine separation of dissolved trace metal species into bioaccessibility-based classes at environmentally-relevant concentrations using asymmetrical flow field-flow fractionation coupled to quadrupole ICP-MS under metal-free, ultra-clean conditions with advanced quality control. *Anal. Chem.* (In preparation).

Neubauer, E., v.d. Kammer, F., Hofmann, T. Using FLOWFFF and HPSEC to determine trace metal-colloid associations in wetland runoff. *Wat. Res.* **47**, 2757-2769 (2013).

Tables and Figures

Table S1. Location of the snow samples shown in Fig. S1, with GPS coordinates and distance from the midpoint between two upgraders.

Site ID	Latitude	Longitude	Distance from midpoint (km)
MIL	56° 55'50.4" N	111° 28'30.3" W	11.0
JPH4	57° 6'44.10" N	111° 25'24.42" W	12.4
McK	57° 13'42.4" N	111° 42'00.8" W	24.9
McM	56° 37'40.4" N	111° 11'39.1" W	48.7
ANZ	56° 28'19.08" N	111° 2'33.66" W	68.4

Table S2. Accuracy and precision of lead (Pb) and thorium (Th) concentrations in NIST 1640a (National Institute of Standards and Technology) and SPS-SW2 (LGC standards) along with the LODs and LOQs measured using ICP-QMS (iCAP Qc).

**SRM**	**NIST 1640a**		**SPS-SW2**	
Trace metal	Lead (Pb)	Thorium (Th)	Lead (Pb)	Thorium (Th)
LOD (ng L^{-1})	0.08	0.01	0.08	0.01
LOQ (ng L^{-1})	0.4	0.04	0.4	0.04
Certified values (µg/L)*	**12.0**	**NA**	**25.0**	**2.5**
SD	**0.0**		**0.1**	**0.0**
SRM (µg/L, n = 3)	10.9		23.5	2.5
Accuracy (%)	91.1		93.8	100.0
Precision (%)	5.8		3.7	4.9
SRM ÷ 10 (µg/L, n = 3)	1.1		2.7	0.3
Accuracy (%)	93.1		106.7	107.4
Precision (%)	2.0		5.2	6.6
SRM ÷ 100 (µg/L, n = 3)	0.1		0.2	0.0
Accuracy (%)	90.6		90.4	88.2
Precision (%)	2.7		5.4	8.7

* Bold values: informational purposes only

Table S3. Accuracy and precision of lead (Pb) and thorium (Th) concentrations in IAEA/SL-1 and IAEA Soil 7 SRMs (International Atomic Energy Institute).

Trace metal	SRM	Certified value ($\mu g\ g^{-1}$)	Measured value ($\mu g\ g^{-1}$)	Accuracy (%)	Precision (%)
Lead (Pb)	IAEA/SL-1 (n = 3)	37.7	41.0	108.9	2.4
	IAEA Soil 7 (n = 3)	60	66	109.2	4.3
Thorium (Th)	IAEA/SL-1 (n = 3)	14	13.7	97.9	3.4
	IAEA Soil 7 (n = 3)	8.2	6.9	84.6	7.8

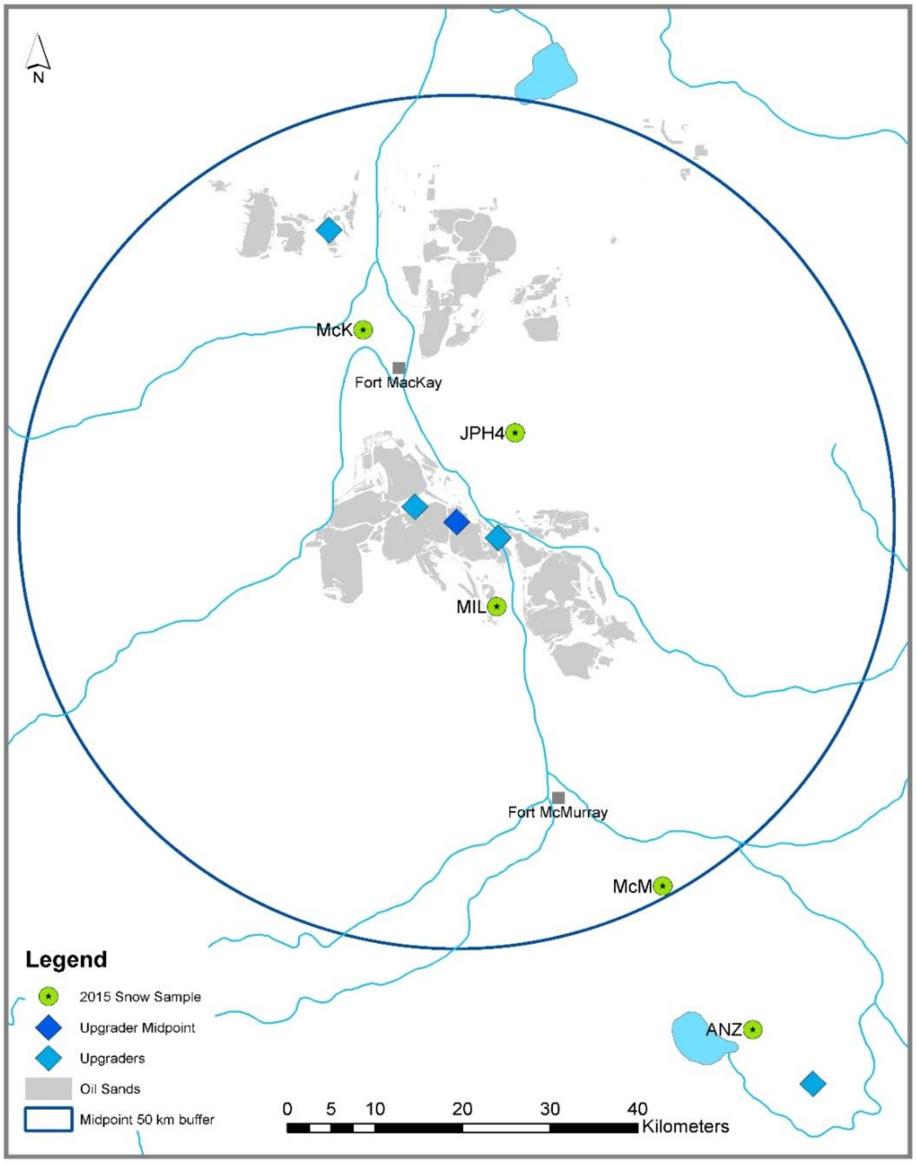

Figure S1. Location of the 5 snow samples, namely McK, JPH 4, MIL, McM, and ANZ collected from the study area. Map also shows the industrial zone, open pit mines + tailings ponds (grey area), location of the bitumen upgraders (blue squares) and the midpoint (5 km from each upgrader) used to measure the distance of the sampling locations. The details of the sampling locations such as latitude, longitude and the distance from the midpoint are given in Table S1. The map is created using ArcGIS Desktop (ESRI 2011: Release 10.3. Redlands, CA: Environmental Systems Research Institute, http://www.esri.com/software/arcgis/arcgis-for-desktop) by taking the basemaps and reference layers information through basemap imagery (Source: Esri, DigitalGlobe, GeoEye, i-cubed, USDA, USGS, AEX, Getmapping, Aerogrid, IGN, IGP, swisstopo, and the GIS User Community).

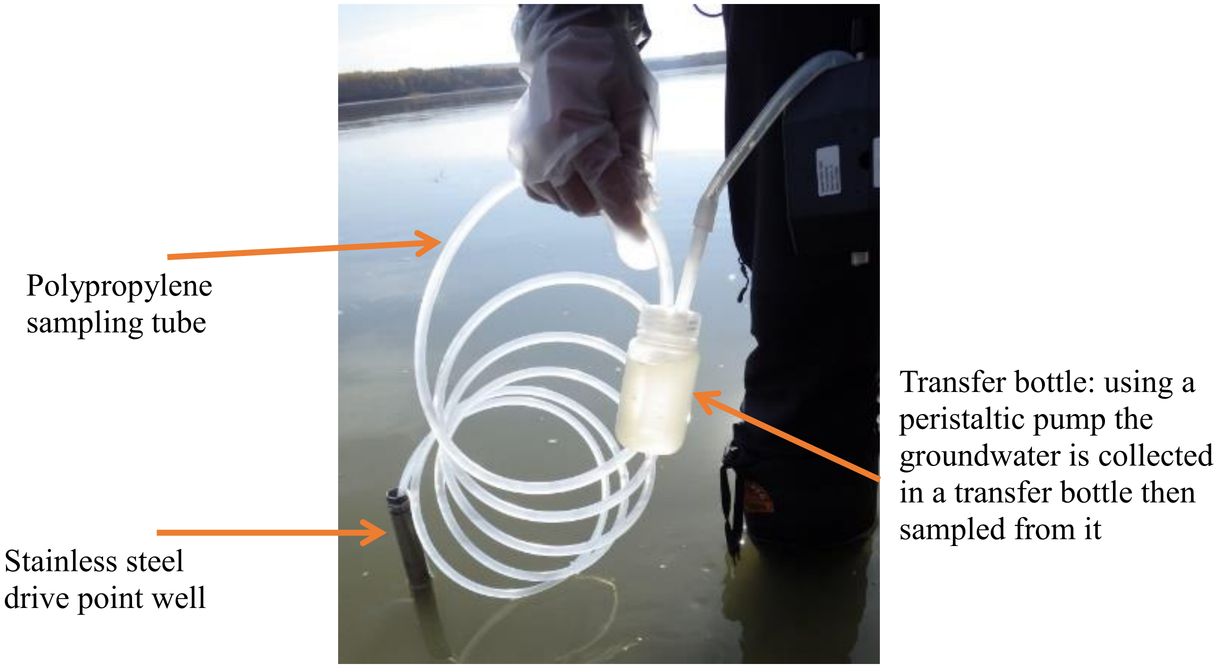

Figure S2. Groundwater sampling scheme designed to minimize contamination.

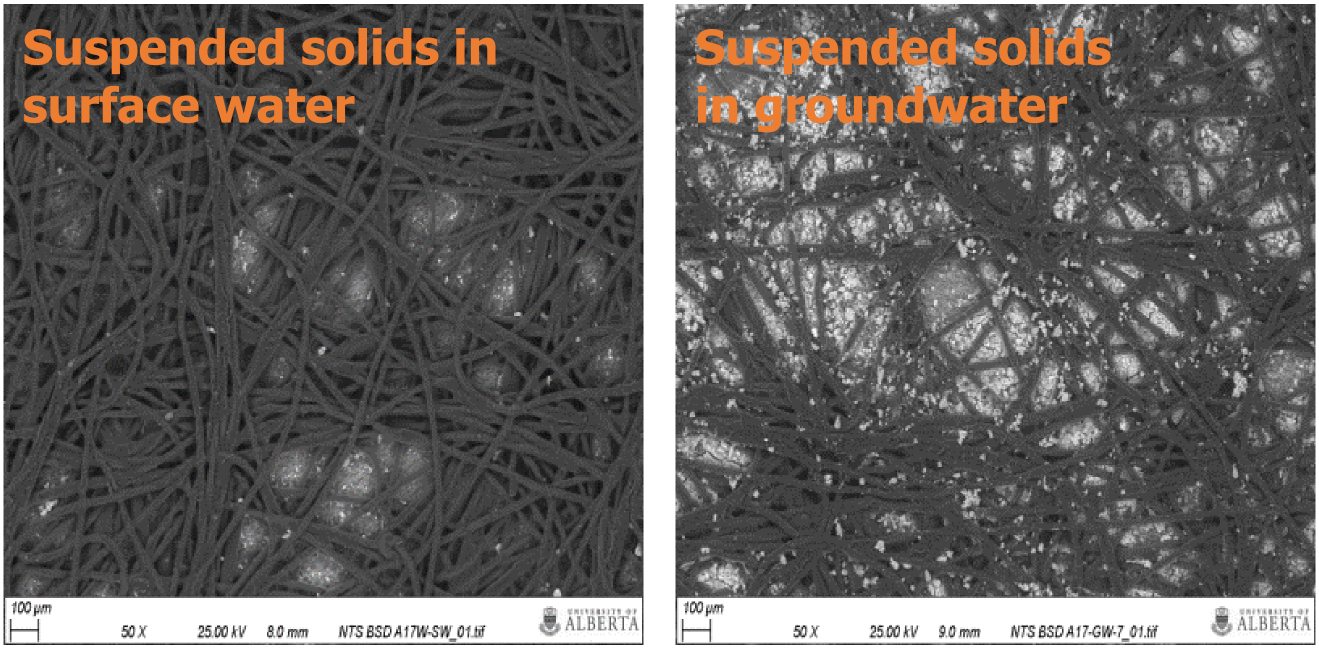

Figure S3. SEM images showing suspended solids (> 0.45 μm) on the filter membranes. The groundwater samples contained more particles than the surface waters.

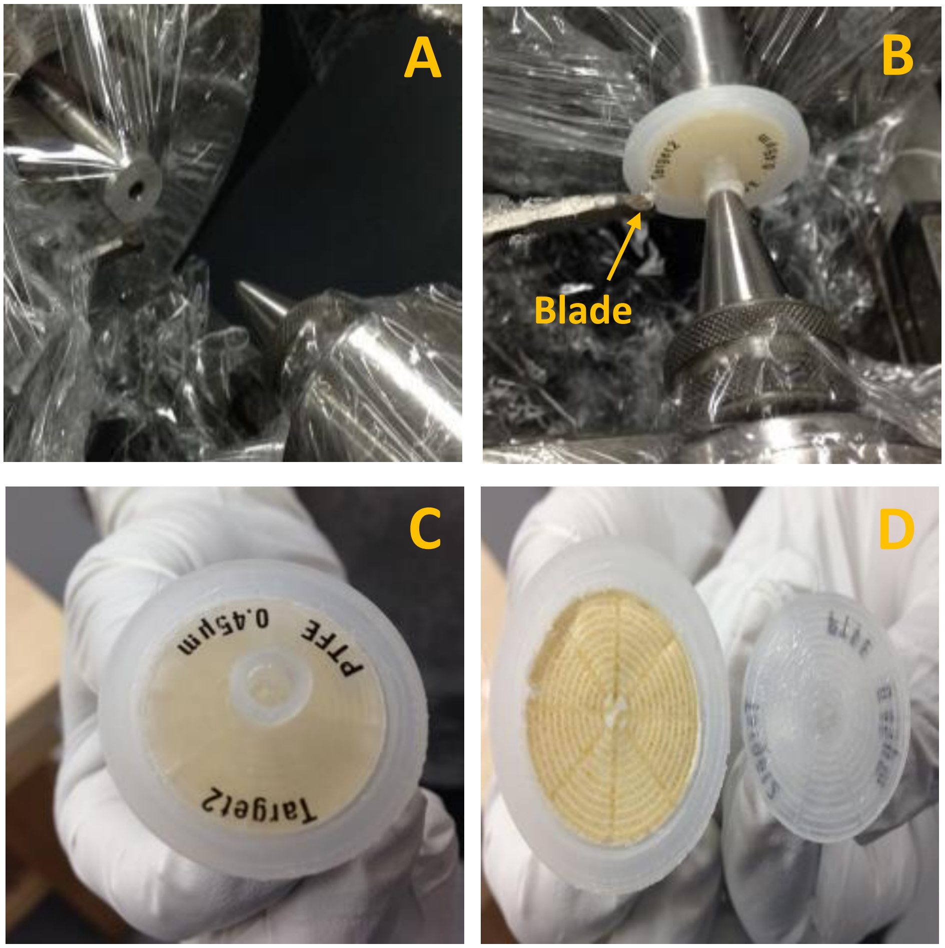

Figure S4. Illustration of the setup devised to precisely cut and open the filter discs to recover the filter membrane containing the suspended sediments (> 0.45 micron). **A**) mechanical set up to cut the filter disc using a precision lathe (Schaublin 135, Bevilard, Switzerland), **B**) adjusted filter firmly held and aligned with blade to cut the filter housing only, **C**) filter after cutting, **D**) filter membrane containing the suspended sediments ready to be picked up without any particle loss.

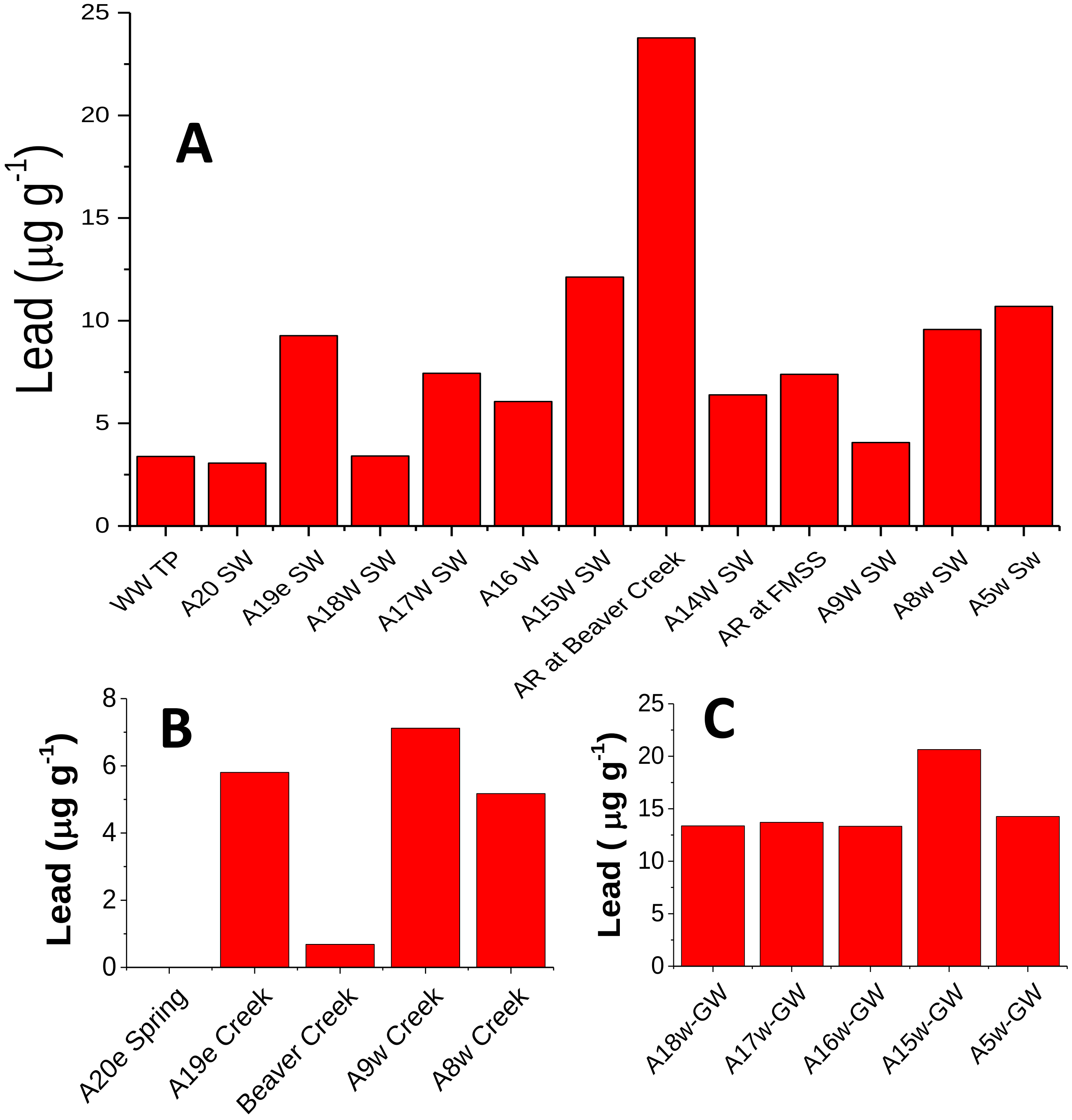

Figure S5. Lead concentrations in the suspended solids in (**A**) the main stem of the AR, (**B**) tributaries and (**C**) groundwater. No sample was collected from A20e spring.
